# The first generation of a BAC-based physical map of *Brassica rapa*

**DOI:** 10.1186/1471-2164-9-280

**Published:** 2008-06-12

**Authors:** Jeong-Hwan Mun, Soo-Jin Kwon, Tae-Jin Yang, Hye-Sun Kim, Beom-Soon Choi, Seunghoon Baek, Jung Sun Kim, Mina Jin, Jin A Kim, Myung-Ho Lim, Soo In Lee, Ho-Il Kim, Hyungtae Kim, Yong Pyo Lim, Beom-Seok Park

**Affiliations:** 1 Brassica Genomics Team, National Institute of Agricultural Biotechnology, Rural Development Administration, 225 Seodun-dong, Gwonseon-gu, Suwon 441-707, South Korea; 2 Macrogen, 60-24 Gasan-dong, Geumcheon-gu, Seoul 153-023, South Korea; 3 National Instrumentation Center for Environmental Management, Seoul National University, San 56-1, Sillim-dong, Gwanak-gu, Seoul 151-921, South Korea; 4 Department of Horticulture, Chungnam National University, 220 Kung-dong, Yusong-gu, Daejon 305-764, South Korea; 5 Department of Plant Science, College of Agriculture and Life Sciences, Seoul National University, San 56-1, Sillim-dong, Gwanak-gu, Seoul 151-921, South Korea

## Abstract

**Background:**

The genus *Brassica *includes the most extensively cultivated vegetable crops worldwide. Investigation of the *Brassica *genome presents excellent challenges to study plant genome evolution and divergence of gene function associated with polyploidy and genome hybridization. A physical map of the *B. rapa *genome is a fundamental tool for analysis of *Brassica *"A" genome structure. Integration of a physical map with an existing genetic map by linking genetic markers and BAC clones in the sequencing pipeline provides a crucial resource for the ongoing genome sequencing effort and assembly of whole genome sequences.

**Results:**

A genome-wide physical map of the *B. rapa *genome was constructed by the capillary electrophoresis-based fingerprinting of 67,468 Bacterial Artificial Chromosome (BAC) clones using the five restriction enzyme SNaPshot technique. The clones were assembled into contigs by means of FPC v8.5.3. After contig validation and manual editing, the resulting contig assembly consists of 1,428 contigs and is estimated to span 717 Mb in physical length. This map provides 242 anchored contigs on 10 linkage groups to be served as seed points from which to continue bidirectional chromosome extension for genome sequencing.

**Conclusion:**

The map reported here is the first physical map for *Brassica *"A" genome based on the High Information Content Fingerprinting (HICF) technique. This physical map will serve as a fundamental genomic resource for accelerating genome sequencing, assembly of BAC sequences, and comparative genomics between *Brassica *genomes. The current build of the *B. rapa *physical map is available at the *B. rapa *Genome Project website for the user community.

## Background

The genus *Brassica *is one of the most important vegetable crop genera in the world because it contributes to human diet, condiments, animal feed, forage, and edible or industrial oil. Many cultivated species of *Brassica *are also increasingly recognized as good sources of healthy metabolites such as vitamin C, soluble fiber, and multiple anti-cancer glucosinolate compounds including diindolylmethane and sulforaphane [1]. In addition, current emphasis on rapeseed oil as a biofuel or a renewable resource for industry worldwide makes *Brassica *a good target of metabolic engineering.

The close phylogenetic relationship between the *Brassica *species and model plant *Arabidopsis thaliana *predicts that the knowledge transfer from *Arabidopsis *for *Brassica *crop improvement would be straightforward. However, the complex genome organization of the *Brassica *species as a result of multiple rounds of polyploidy and genome hybridization makes the identification of orthologous relationships of genes between the genomes highly difficult. In particular, comparative genomics study of Flowering Locus C region between *B. rapa *and *A. thaliana *genomes revealed that the *Brassica *genome triplicated 13 to 17 million years ago very soon after divergence from the *Arabidopsis *lineage. A following extensive interspersed gene loss or gain events and large scale chromosomal rearrangements including segmental duplications or deletions in the *Brassica *lineage complicated the orthologous relationships of the loci between the two genomes [2]. Hybridization between *Brassica *species is another source of the *Brassica *genome complexity. The interspecific breeding between three diploid *Brassica *species, *B. rapa *(AA genome), *B. nigra *(BB genome), and *B. oleracea *(CC genome), resulted in the creation of three new species of allotetraploid hybrids *B. juncea *(AABB genome), *B. napus *(AACC genome), and *B. carinata *(BBCC genome) [3]. Thus, investigation of the *Brassica *genome provides substantial opportunities to study the divergence of gene function and genome evolution associated with polyploidy, extensive duplication and hybridization.

Several crop *Brassica *species have had their genomes characterized in-depth. With favorable genetic attributes, *B. rapa *has been selected as a model species representing the *Brassica *"A" genome and is the focus of multinational genome projects. The early fruits of investigation with this well-characterized genome are evident in the recent advance in our understanding of *Brassica *"A" genome structure and evolution [2,4-7]. Linkage maps have been constructed for *B. rapa *ssp. *pekinensis *cv. *Jangwon *[4], cv. *VCS *(Kim et al., unpublished our data), and cv. *Chiifu *[5]. These genetic maps with associated markers and comparative genomics study have enabled the identification of quantitative trait loci (QTL) for club root resistance and flowering time. Large EST databases are publicly available and a 24 K oligo microarray has been developed and used to examine the transcriptome profile of *B. rapa *[8]. More than 127,000 Bacterial Artificial Chromosome (BAC) end sequences and about 580 seed BAC sequences of phase 2 or 3 are also available at the National Center for Biotechnology Information (NCBI) database. In parallel to these activities, international programs are collaborating to characterize the *Brassica *"A" genome at the whole genome sequence level through a BAC-by-BAC sequencing approach [9].

A crucial component of successful genome sequencing activity with the BAC-by-BAC strategy is the availability of a genome-wide, BAC-based physical map [10]. To date, the utility of a physical map has been reported by major genome sequencing projects of human [11], *A. thaliana *[12], *Oryza sativa *[13], and *Medicago truncatula *[14]. These physical maps were constructed with a combination of restriction-enzyme digested BAC fragments fingerprinting on agarose gels and assembly of the fingerprints by means of FingerPrinted Contigs (FPC) software package [15]. The agarose method has been successful, but it has limited throughput because of the need for human band calling. This is a time-consuming process requiring ample skill even when using image software [16]. Another disadvantage of the agarose method is that few large fragments are generated, and they are difficult to size. Bands manually selected using the agarose method can often lead to a poor map [17,18]. Fluorescence-labeled fingerprinting methods using DNA sequencing gel [19,20] or capillary electrophoresis [21,22] are alternative methods that have been developed to make larger and more accurate contigs with increased throughput. Fluorescence-labeled capillary electrophoresis methods include the 3-enzyme method [22] and the High-Information Content Fingerprinting (HICF) methods which use type IIS restriction enzyme [16] or the SNaPshot labeling technique [21,23-25]. These methods facilitate improved physical map construction both in terms of throughput and quality of fingerprinting compared to the agarose method due to their automatic workflow and higher resolution [17,22]. However, an increase in the number of enzymes and labeling colors in the HICF method can give partial digestion, star activity, and low labeling efficiency [23]. Accordingly, several whole-genome HICF assembly maps have been built for small fungi genomes [23,24] as well as for large genomes of maize [16] and catfish [25].

*Brassica rapa *has a haploid genome size of 550 megabase pairs (Mb) [26]. Here we report the first genome-wide, BAC-based SNaPshot physical map of the *Brassica *"A" genome. To build a physical map, we have fingerprinted about 99,000 BAC clones by the HICF method using an ABI SNaPshot labeling kit and constructed a BAC clone contig map by means of FPC v8.5.3. Sequence-tagged site genetic markers incorporated in the genetic map anchored the euchromatic portion of the physical map to chromosomal loci. The resulting physical map allows facilitated selection of BAC clones for the *B. rapa *whole genome sequencing effort.

## Results and discussion

### BAC library source and fingerprinting

Construction of a physical map for a genome that has evolved through polyploidy, extensive genome duplication or hybridization presents robust challenges to genome analysis. Successful contig build of the *B. rapa *genome relies on the quality and availability of deep-coverage large insert genomic libraries. Three large-insert BAC libraries of *B. rapa *ssp. *pekinensis *cv. *Chiifu *are available in the public sector providing >34-fold genome coverage [7,8]. The first step to construct a physical map is generation of fingerprints representing restriction digests of BAC DNA using efficient techniques [20,27]. We have chosen the HICF fingerprinting method based on its well-established format with a commercially available SNaPshot labeling kit (ABI) and increased throughput using the ABI 3730 xl sequencer [17,21]. A total of 99,456 BAC clones (~22.5× coverage) from the three independent libraries were fingerprinted by digestion with five restriction enzyme combinations (*Eco*RI, *Bam*HI, *Xba*I, *Xho*I, and *Hae*III) followed by SNaPshot reagent labeling of four colors at the 3' ends of the restriction fragments and sizing on the ABI 3730 xl (Table 1). The size of DNA fragments from the capillary fingerprinting chromatograms was collected by GeneMapper. There was an average of 114 restriction fragments produced per BAC clone. The average size of the band was calculated as 1.09 kb with average insert size of BAC clones at 124 kb. The fingerprint data was then imported to GenoProfiler [28] to change data format suitable for FPC analysis. Of these fingerprints, 5,767 (5.8%) were removed from the data set due to no insert clones, failure in fingerprinting, clones having fewer than 50 bands or more than 200 bands in the range of 50–500 bp, or cross-contamination. Thus, a total of 93,689 clones (94.2%) were successfully fingerprinted to be used for contig assembly.

**Table 1:** Characteristics of the three source BAC libraries of *Brassica rapa *ssp. *pekinensis *cv. *Chiifu *that were used in the HICF map.

Libraries^a^	Genomic DNA partially digested with	Average insert size (kb)	No. of 384 plate	No. of BACs	Average no. of valid bands per clones^b^	Genome coverage^c^	No. of BACs with successful fingerprints	No. of BACs with repetitive sequences
KBrH	*Hin*dIII	125	KBrH001-147	56,448	124	12.9×	53,443	16,402
KBrB	*Bam*HI	126	KBrB001-096	36,864	104	8.5×	34,371	9,604
KBrS	*Sau*3AI	100	KBrS001-016	6,144	94	1.2×	5,875	215

Total		124	259	99,456	114	22.5×	93,689	26,221

^a^For details of the BAC libraries, see [7] and [8].

^b^Valid bands are those in the range of 50~500 bp.

^c^Genome coverage was estimated based on the haploid genome equivalent of *B. rapa *as 550 Mb.

### Contig assembly

With BAC fingerprints, the creation of a physical map of a eukaryotic genome is a three-step process. First, the fingerprints are assembled into contigs, which are accurately ordered contiguous overlapping clone sets [29]. Second, the contigs are anchored on the genetic map to accurately represent the true order [30,31]. Third, questionable contigs are broken to increase contig reliability or contigs associated with adjacent regions of the genome are fused to organize big contigs [32]. Genome duplication, repetitive sequence blocks, questionable clones (Q clones), and/or fingerprinting error complicate these steps and can result in contigs containing false-positive overlaps of clones [16,29]. Therefore, as a prelude to developing a reliable physical map of *B. rapa*, it is worth discarding low quality or problematic data before the fingerprint assembly to avoid chimeric contigs. Moreover, the eliminated clones can later be placed back onto the physical map after the contig merger is completed [21]. In the three *B. rapa *BAC library sources, up to 29% clones were estimated to contain centromeric or pericentromeric repetitive sequences [6]. To screen out the clones having heterochromatic repetitive sequences before contig assembly, we removed 26,221 clones (28.0%) containing centromeric repetitive sequences (CentBr and CRB) at least in one end or pericentromeric repetitive sequences (PCRBr, 5S, and 25S rDNA) in both ends based on BLASTN search of BAC end sequences (Table 1). Thus, a total of 67,468 BAC clone fingerprints with an average band size of 1.39 kb (Table 2), equivalent to 15.2× of the *B. rapa *genome, were finally converted into the FPC database. Of these 67,468 clones, 37,041 (8.4×) were from the *Hin*dIII library, 24,767 (5.7×) from the *Bam*HI library, and 5,660 (1.0×) from the *Sau*3AI library.

**Table 2:** Summary of the *B. rapa *physical map autobuild produced from assembly of the 67,468 BAC clones.

Build^a^	Contigs	Avr. contig length (kb)^b^	Longest contig (kb)	Genome coverage	Physical length (Mb)^b^	Q clones (%)	No. of contigs of different sizes					Singletons
								
							≥ 100	99-50	49-25	24-10	<10	
Initial 1e-45	4,726	208	7,596	1.8×	985	6,376 (9.5)	9	34	251	892	3,540	25,041
Merge 1e-40	4,057	230	5,548	1.7×	935	6,457 (9.6)	10	64	318	800	2,865	23,977
Merge 1e-30	3,001	287	7,329	1.6×	860	6,927(10.3)	24	126	384	606	1,861	21,351
Merge 1e-20	1,801	421	8,686	1.4×	759	8,832(13.1)	82	182	299	370	868	17,086
Merge 1e-15	1,417	512	9,390	1.3×	725	10,135(15.0)	111	177	241	269	619	14,001

Each HICF assembly was performed starting with a complete build, followed by iteration of the Dqer, end-merge, and singleton-merge routines by means of FPC v8.5.3.

^a^Additional Dqer, end-merge, and singleton-merge routines at 1e-35 and 1e-25 are not shown.

^b^Each fingerprint band was estimated to represent an average of 1.39 kb. It was estimated by the average insert size of the BAC clones (124 kb, Table 1) divided by the total number of valid bands of 67,468 BAC clones (6,005,758 bands) used for the map contig assembly.

To assemble the physical map contigs of the *B. rapa *genome from BAC fingerprints, we used the program FPC v8.5.3. Before contig assembly, a series of tests were performed to determine the FPC parameter suitable for contig assembly of the full data set. Contig build at high stringency prevents chimeric joining of duplicated regions, whereas starting builds at low stringency results in maps with larger contigs that encompass more genome space [16]. Thus, the best approach should rely on the structural characteristics of a target genome. The automatic contig build using a randomly chosen data set was tried with different cutoff values from 1e-40 to 1e-80. Based on the preliminary test, the initial cutoff value was chosen to be 1e-45. The initial parameter is reasonably stringent because the contigs generated at this cutoff value included up to 70% of the clones with less than 10% questionable clones (Q clones) which can cause chimeric assembly. Of course, assembly at higher stringency improved the build by reducing Q clones but contig coverage reduced significantly. For example, contig build at 1e-70 included only 40% of the fingerprints in contigs and left 60% as singletons. Based on this analysis, we assembled the physical map contigs in three steps. First, a cutoff value of 1e-45 was used for automatic contig assembly. Second, the "DQer" function was used to break up Q contigs (contigs containing more than 10% of Q clones) from the initial builds. Third, the remaining contigs were end-merged by "End to End" function and then singletons were added to the end of contigs by "Singles to End" function at 6 successively lower cutoffs, starting at 1e-40 and terminating at 1e-15. At each round, additional "DQer" was used to break up all bad contigs containing more than 15% Q clones (Table 2). As a result, the first contig build resulting from automatic assembly and DQer contained 4,726 contigs assembled with 42,427 (63%) clones but 25,041 (37%) clone fingerprints remained as singletons. Following an iterative process of consecutive FPC functions, "End to End", "Singles to End", and "Dqer", each successive round contributed nicely to a decrease in the contig number, singleton number, and genome coverage but to an increase in average contig length (Table 2). It is obvious that merger of singletons into the assembly is responsible for most of the increase in the number of Q clones in the map [16]. However, Table 2 shows that only ~34% of singletons integrated into the end of the contigs contributed to the increase of Q clones in the build. This result suggests that many clones that remained as singletons at the initial stringency cutoff are not just because their fingerprints were low quality but because they may come from regions of low coverage. If this is true, the BAC libraries we used would not deeply cover the whole *B. rapa *genome. An unsatisfactory aspect of this assembly is its large number of Q clones (Table 2). The Q clones in this assembly corresponded to 15% of the clones. This is a bigger proportion than the cases reported from catfish (7.3%) [25] and maize (11%) [16]. A large number of Q clones may result from fingerprinting error due to partial digestion, star activity, or low labeling efficiency. Though we removed the fingerprints containing centromeric repeat sequences, the remaining dataset still included highly repetitive DNA sequences. If repetitive sequences significantly affect contig assembly, deep contigs (too many clones assembled in a small region) can be made. The impact of repetitive DNA sequences on the contig assembly has been estimated. Of the 1,417 contigs, three were found to be deep contigs. Chloroplast DNA can be a source of deep contig assembly [33]. However, Blast analysis of *B. rapa *chloroplast sequence against BAC-end sequences from the deep contigs suggested that these deep contigs may be derived from *B. rapa *genomic DNA. These three deep contigs included 71–84% of the clones as Q clones, which contribute to ~48.3% of all Q clones in the initial build. Thus, when we kill three deep contigs of the initial build due to false positive overlaps, the Q clones in the remaining 1,414 contigs correspond to 7.7% of the whole clones.

The initial build, named *B. rapa *physical map Build 1, has 1,417 contigs with an average length of 512 kb covering 725 Mb, 1.3× coverage of the genome. The total coverage of the physical contigs suggests that most contigs are not sufficiently overlapping and the gaps between the contigs need to be closed by additional fingerprinting. However, with our current assembly, more fingerprinting of the same libraries would not be effective in increasing coverage of the contigs and closing the gaps efficiently, because a higher proportion of the BAC clones are covering repetitive sequence regions and some regions of the genome could be poorly represented in those libraries generated by restriction enzyme digestion. For this reason, we will add more fingerprint data from a randomly sheared BAC library that is under construction, and will develop a new contig build.

### Validation of contigs and manual editing

**Figure 1:**
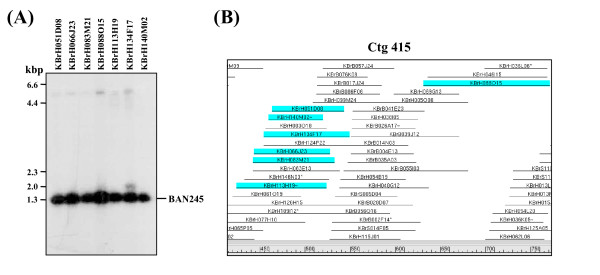
**An example of correspondence between a single locus RLFP marker, BAN245, and its corresponding contig containing all positive BAC clones**. (A), Southern blot analysis of seven positive BAC clones picked from the KBrH library. *Hin*dIII digestion of BAC DNA followed by BAN245 hybridization shows a single hybridization band for the BAN245 marker. (B), view of fingerprint contig containing all seven positive BAC clones for the BAN 245 marker. The blue highlighted clones are those screened by Southern hybridization.

**Figure 2:**
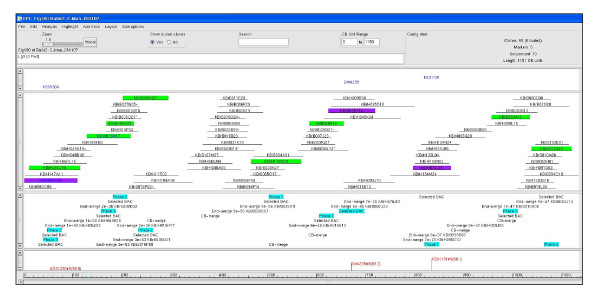
**An example of a BAC physical contig anchored to the R9 chromosome of the *B. rapa *genome**. This contig consists of 68 BAC clones from three source BAC libraries (Table 1) and is estimated to cover approximately 1.3 Mb. The clones prefixed with KBrH were from the *Hin*dIII library, with KBrB from the *Bam*HI library, and with KBrS from the *Sau*3AI library. This contig was anchored to the region around 86–91 cM of the R9 genetic map using two SSR markers, KS31203 and KS31191, and one RFLP marker, BAN235. The violet-highlighted clones represent the corresponding BAC clones containing the respective DNA markers. All the highlighted BAC clones are in the genome sequencing pipeline and their sequencing phases are indicated.

Several different approaches were used to evaluate the reliability of the *B. rapa *contig assembly. First, marker anchors have been developed as an effective tool to validate contig structure and orientation. We analyzed whether positive BAC clones of single locus RFLP markers were assembled into the same segment of a contig. For example as shown in Figure 1, a total of seven positive BAC clones were identified through a *Hin*dIII BAC library screen using a single locus BAN245 marker designed from a hydrolase gene (Fig. 1A). FPC database search showed that six of the positive clones were assembled into the same segment of contig 415, and one clone was located very close to the others on the consensus band (CB) map (Fig. 1B). Marker anchors strongly supported proper assembly of contigs. We anchored 187 contigs on an existing genetic map [4] using 315 genetic markers (Table 3 and Table S1 in additional file 1). Among the 187 contigs containing BAC clones associated with framework genetic markers, 37 contigs having at least two marker anchors were selected to validate the contig build. The framework markers displayed close genetic linkage for contigs. Even nine questionable contigs (greater than 10 Q clones per contig) of the 37 contigs showed nice anchoring of the marker pairs on the genetic map. Figure 2 presents an example of contig validation by mapping, in which a contig spanning the region of 86–91 cM of linkage group R9 was examined. A single locus RFLP marker, BAN235, designed from a pectinesterase (PE) gene expressed in anther was first used to screen the *Hin*dIII library, and three positive BAC clones (KBrH016E13, KBrH059J05, and KBrH071P14) were identified at high stringency. An FPC database search detected the corresponding contig containing the positive clones. Contig 180 consisted of 68 BAC clones and was 1.3 Mb in size. Two SSR markers, KS31203 and KS31191, were designed from the BAC clones KBrH001H24 and KBrH076J01, respectively, which were found at both ends of the contig. Genetic mapping of the SSR markers showed close genetic linkage on linkage group R9, consistent with clone orders in the contig. This result was supported by sequence analysis of the selected BAC clones. BAC sequence analysis of 11 selected clones in this contig successfully generated two overlapping sequence blocks in accordance with the genetic mapping result. Additional mapping and BAC sequencing enabled merger of contig 180 with five adjacent contigs to make a big contig extended to 3.1 Mb in size (data not shown).

**Table 3:** Summary of sequence-tagged site genetic markers used for contig integration into the *B. rapa *genetic map.

Total number of markers used	315
Total number of positive clones	306
Positive clones in contigs	234
Positive singleton clones	72
Number of markers in contigs	242
Number of markers in singletons	73
Number of contigs containing genetic markers	187
Contigs containing one genetic marker	150
Contigs containing more than one genetic markers	37

**Figure 3:**
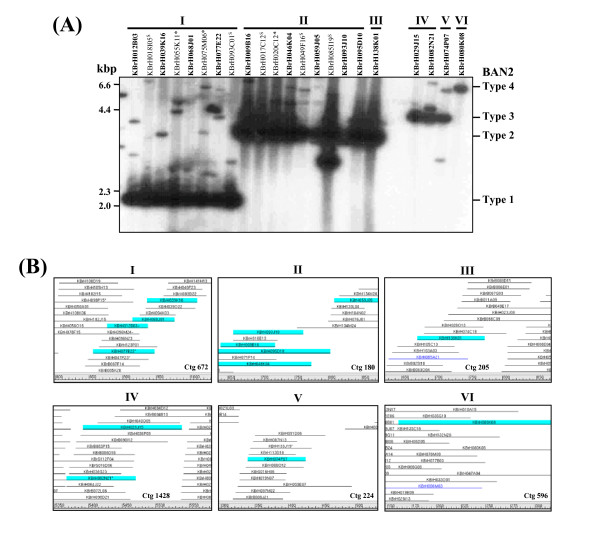
**An example of correspondence between four different types of multiple loci genetic marker, BAN2, and their distinct physical contigs containing corresponding BAC clones**. (A), Southern blot analysis showing four different hybridization patterns of BAN2 markers for 22 BAC clones picked from the KBrH library. I-VI represent the grouping of the BAC clones according to their main hybridization bands from *Eco*RV digestion followed by BAN2 hybridization and fingerprint contig information. *Three clones were excluded from the fingerprint assembly due to failed fingerprinting. ^s^Five clones remained as singletons after contig assembly. (B), view of six different fingerprint contigs containing the corresponding groups of 14 BAC clones for respective marker types. The blue highlighted clones are those screened by hybridization.

As a second validation, a grouping of a multigene family was examined to determine if clones containing paralogous genes would be correctly assembled in the HICF map. As shown in Figure 3, the contigs spanning the regions containing the pectinesterase gene family members were investigated. At least 14 members of the PE gene family were identified from a *B. rapa *EST database search. Screening of the *Hin*dIII library using a RFLP marker BAN2 designed from a PE gene identified 22 positive BAC clones. Southern blot analysis of the 22 clones by *Eco*RV digestion and hybridization with the BAN2 probe grouped the clones into at least four different types according to shared main bands (Fig. 3A). We analyzed the contig assembly of 19 clones successfully fingerprinted from the 22 positive BAC clones. HICF assembly of the 19 clones resulted in grouping of 14 clones in six independent contigs consistent with the observed Southern hybridization pattern (groups I to VI corresponding to contigs 672, 180, 205, 1428, 224, and 596, respectively); the remaining five clones were singletons (Fig. 3B and Fig. S1 in additional file 2). The clones of groups II/III and groups IV/V shared the same main hybridization bands of Type 2 and Type 3, respectively, but they were assembled in separate physical contigs. These results strongly support the assumption that paralogous clones are correctly assembled in independent contigs or remain as singletons in the current build. We found five additional cases of correctly assembled homeologous regions (data not shown).

Finally, the reliability of the assembly has been confirmed by the results of ongoing genome sequencing of *B. rapa*. Integration of physical contigs into the genetic loci identified a conflict between anchors of sequence-tagged site markers. Contig 166 was found to be assembled by a false joining. Two of the markers, KS50140 and KR50161, anchored on this contig belonged to linkage group R3 but KS10551 marker was assigned to R9. We checked the CB maps of the fingerprint order of this contig and found that two independent contigs were joined by end merge at 1e-25. To further test that the merger of two structurally related genome clusters at low stringency generated a chimeric contig, we analyzed nine BAC clone sequences of this contig which were included in our genome sequencing pipeline for chromosomes R3 and R9. Sequence analysis demonstrated that seven BAC clones, associated with markers KS50140 and KR50161, assembled with one sequence scaffold of chromosome R3, whereas two BAC clones, associated with marker KS10551, merged into an existing sequence block of chromosome R9 (data not shown). Based on these results, we manually broke up this contig into two independent contigs, contig 87 and contig 190, by splitting at the weak point of the CB map. A similar conflict was found in one of the deep contigs previously mentioned. Due to complex fingerprint information and many Q clones originating from repetitive sequences, we killed this contig rather than split it. Since our analysis included only a few contigs, overall reliability of current contig build is limited. However, this validation study provided a contig assembly error estimated at 5%, in agreement with the previous reports of maize (4%) [16] and catfish (5%) [25], in which the HICF method was used. As of December 2007, chromosome sequencing of R3 and R9 on our sequencing pipeline have generated 21 and 27 sequence scaffolds which cover approximately 23 Mb and 26 Mb, respectively. Sequence analysis of the scaffolds provided validation of at least 204 contigs (data not shown).

With the results of contig evaluation, incorporation of genetic marker information, and BAC sequencing, manual editing of the initial contig build yielded Build 2. As shown in Table 4, Build 2 consists of 1,428 contigs spanning 717 Mb. Interestingly, Blast analysis of BAC-end sequences against our *B. rapa *EST database showed that 1,227 contigs (86%) estimated to span ~616 Mb are delimited to cover presumably gene-rich regions. We note that removal of heterochromatic BAC clones before assembly significantly enriched the euchromatic contigs in the build. Of practical importance, integration of a physical map into a genetic map enabled the positioning of 242 gene-rich contigs to specific locations on 10 linkage groups providing seeds for the current genome sequencing effort. The extent of the contigs associated with genetic loci is ~160.7 Mb, or 29% of the total genome. As we showed, marker integration is a powerful tool to resolve questions on the physical map. During marker integration, we found that hybridization-based RFLP markers occasionally misassigned corresponding BACs. The possible origin of this misassignment includes highly conserved duplicated genome segments or recently evolved gene paralogs that have distinct locations in the triplicated *B. rapa *genome. Therefore, sequence-tagged site markers rather than RFLP markers are the preferable marker type for accurate BAC anchoring on the *B. rapa *genome. Additional genetic mapping, further integration of the genetic and physical maps based on sequence-tagged site markers, and the progress of genome sequencing will improve build quality and ultimately determine which contigs are substantially correct, contain merged homeologous regions or are otherwise incorrect.

**Table 4:** Summary of the *B. rapa *physical map Build 2.

Number of clones fingerprinted	99,456
Number of clones with successful fingerprints	93,689
Number of clones used for the map construction	67,468
Number of singletons	14,816
Number of contigs	1,428
>200 clones	32
101–200 clones	73
51–100 clones	176
26–50 clones	244
10–25 clones	284
3–9 clones	380
2 clones	239
Physical length of the contigs (Mb)	717
Number and length of contigs anchored to chromosome^a^	242 (160.7)
R1	18 (13.6)
R2	19 (13.2)
R3	57 (36.5)
R4	6 (2.0)
R5	18 (9.0)
R6	17 (13.8)
R7	14 (12.7)
R8	17 (12.2)
R9	66 (39.7)
R10	10 (8.1)

^a^Length of physical contigs anchored to each chromosome is represented as Mb in parenthesis.

## Conclusion

We constructed a genome-wide BAC contig map of the *B. rapa *genome. This is the first whole genome physical map representing the *Brassica *"A" genome. As of August 7, 2007, *B. rapa *physical map Build 2 can be accessed by the user community by means of WebFPC. The physical map created in this study contributes to a fundamental understanding of the *Brassica *"A" genome structure and function as well as to the ongoing genome sequencing project as a resource for facilitating BAC selection and assembly of the genome sequence. With the goal of constructing a sequence-ready physical map, the current anchors of the contig assembly provide 242 seed points which are being extended by the BAC-by-BAC genome sequencing approach of the Multinational *Brassica *Genome Sequencing Project (MBGSP). In addition, the map will serve as a platform to accelerate development of *Brassica *comparative genomics by merging data collected from *B. oleracea*, a model of *Brassica *"C" genome (Paterson and Pires, personal communication). Efforts continue to improve the map by adding fingerprints from a randomly sheared BAC library, additional genetic mapping, and hybridization using overgo probes. All data presented in this paper with updates are available through the *B. rapa *Genome Project website [8].

## Methods

### Source BAC Libraries

Three BAC libraries used in this study were constructed using partial digests with three different restriction enzymes, *Bam*HI, *Hin*dIII, and *Sau*3AI, as described previously (Table 1) [7,8]. The DNA source for the BAC libraries was from the reference plant line of *B. rapa *ssp. *pekinensis *cv. *Chiifu*. Nearly all BAC clones used for fingerprinting were from the *Bam*HI and *Hin*dIII libraries, with a few BACs from the *Sau*3AI library.

### BAC fingerprinting

BAC clones maintained in a 384-well microplate were inoculated in four 96-deep well plates containing 2 ml of 1× LB medium with 12.5 ug/ml chloramphenicol using a Biomek-FX liquid handler (Beckman Coulter, USA). Plates were covered with Airpore gas-permeable plate sealant (Qiagen) and incubated at 37°C for 20–24 hours with continuous shaking at 900 rpm on a BioShaker (Taitek, Japan). BAC DNA was isolated using a modified alkaline lysis method followed by purification. Typically 1 to 1.5 ug of BAC DNA was obtained per BAC clone. Purified BAC DNA was digested with a mixture of five restriction endonucleases, *Bam*HI, *Eco*RI, *Xba*I, *Xho*I, and *Hae*III, for fragmentation. The digested DNA was labeled using ABI PRISM SNaPshot Multiplex kit (ABI No. 4323159) according to the manufacturer's instruction. The fluorescent BAC fingerprinting fragments were resuspended in 10 ul per well of Hi-Di formamide solution and then loaded onto an ABI 3730 xl DNA analyzer with 0.05 ul GeneScan-500 LIZ (ABI No. 4322682, size range from 35 to 500 bp) as an internal size standard.

### Fingerprint data collection and BAC contig assembly

The fingerprint profiles for each BAC clone were collected by GeneMapper v3.7 (ABI) and then converted to a data format suitable for FPC application via GenoProfiler v2.1. Bands ranging from 50 to 500 bp in size were collected for contig assembly. For the data quality control, vector bands and clones failing fingerprinting or lacking inserts were removed manually. In addition, all samples with fewer than 50 band fragments and more than 200 band fragments were also removed. Contig assembly was carried out using FPC v8.5.3 [15] on an HP ML370G5, with two 2.66-GHz Dual-Core Intel Xeon 5150 processors, equipped with a Redhat Enterprise Linux AS 4 platform. FPC parameter was adjusted as described by Luo et al. [21] and Nelson et al. [16] for the HICF technique. Briefly, a series of tests were conducted in which fingerprints of several sets of overlapping clones were compared using different tolerance (from 4 to 6) and cutoff (from 1e-80 to 1e-40) values. On the basis of these tests, tolerance was set at 4 to obtain the 0.4 bp optimal tolerance value determined by Luo et al. [21] for HICF-SNaPshot fingerprinting and the gel length was set at 20,000 bp. An initial Sulston cutoff score of 1e-45 was finally selected to be optimal for contig assembly in order to minimize the number of contigs without overly increasing the number of questionable clones. Contigs with more than 10 Q clones were reassembled by the "DQer" function of FPC. The resulting contigs were merged by "End to End" auto merge function with a minimum of two matching ends. The remaining singletons were merged to the contigs by "Singles to End" function and the "DQer" function was used to finish the process by removing Q clones from the resulting contigs.

### BAC anchoring and manual contig editing

To anchor BAC-based physical contigs to the genetic and cytogenetic maps, 315 sequence-tagged site genetic markers developed from the sequenced BAC clones were used [[4] and Jin et al., unpublished our result]. During BAC anchoring, the contigs showing conflict in the marker-BAC relationship were manually split based on CB map and BES information. Centromeric repetitive sequences (CentBr and CRB), pericentromeric repetitive sequences (PCRBr, 5S, and 25S rDNA), and chloroplast sequence (NCBI accession DQ231548) were analyzed by BLASTN search at cutoff value 1e-10 against BAC end sequence database downloaded from NCBI.

### High-density BAC filter screen and Southern blot analysis

The high-density *Hin*dIII BAC filters were made according to Park et al. [7]. The BAC DNA (50 ng) was digested with *Eco*RV or *Hin*dIII, separated in 1% agarose gel, and transferred onto a nylon membrane (Hybond N^+^, Amersham Pharmacia Biotech) using the standard capillary transfer method. To make RFLP probes, insert DNA of BAN2, BAN235 and BAN245 cDNA clones were amplified by PCR using T3 and T7 primers and then purified by Qiagen gel extraction kit. Probes were labeled using random oligonucleotide priming under the conditions according to the manufacturer's instruction (Megaprime Labeling System, Amersham Pharmacia Biotech). Hybridizations were carried out at 65°C for 24 h with [α-^32^P]-labeled DNA probes. Following hybridization, membranes were washed twice in 2 × SSC and 0.5% SDS for 15 min, followed by 1 × SSC and 0.1% SDS for 20 min, and 0.5 × SSC and 0.1% SDS for 20 min at 65°C. The membranes were exposed to X-ray film for 2–3 days at -80°C with intensifying screens.

## Authors' contributions

J–HM analyzed fingerprints, assembled the physical map, verified contig assembly and wrote the manuscript. S–JK and H–SK developed the BAC DNA extraction and fingerprinting protocols. T–JY, H–SK, JAK, M–HL, SIL, and HK obtained the fingerprints and imposed quality controls on data entering the analysis pipeline. B–SC and SB developed databases and interfaces to display FPC results on the web. JSK and MJ developed markers for contig validation. H–IK and YPL edited the manuscript. B–SP conceived the project and supervised its execution. All authors read and approved the final manuscript.

## Supplementary Material

Additional file 1 Table S1: List of genetic markers used in map integration and their corresponding BAC clones. The data provided represent the correspondence of genetic markers and BAC clones used in integration of genetic and physical maps. Click here for file

Additional file 2 Figure S1. The clone order fingerprints of 19 of 22 BAC clones for the BAN2 marker. This figure shows fingerprinted band image of 19 positive BAC clones of the BAN2 marker. Click here for file
