# Supplementary material for: The first generation of a BAC-based physical map of Brassica rapa
Source: BMC Genomics. 2008 Jun 12;9:280. doi: 10.1186/1471-2164-9-280 (PMC2432078; doi:10.1186/1471-2164-9-280)
Supplement: Additional file 1 — Table S1: List of genetic markers used in map integration and their corresponding BAC clones. The data provided represent the correspondence of genetic markers and BAC clones used in integration of genetic and physical maps. [file 1471-2164-9-280-S1.pdf]

Table S1: List of genetic makers used in map integration and their corresponding BAC clones.

Marker	Corresponding BAC	Contigs	Linkage Group	cM
KS40920	KBrB048F07	Ctg1	R01	0.0
KS41010	KBrB022O03	Singleton	R01	3.8
KS40971	KBrB001A20	Singleton	R01	6.1
KS40990	KBrS004J05	Singleton	R01	8.0
KS40980	KBrB091M07	Ctg2	R01	11.5
KS40870	KBrB092P05	Ctg3	R01	21.3
KS40860	KBrB036M17	Singleton	R01	24.0
KS40840	KBrS004O21	Ctg3	R01	25.2
KS40800	KBrB010F19	Ctg4	R01	36.9
KS40780	KBrB010F06	Ctg4	R01	38.7
KA40500-2	KBrB001M05	Singleton	R01	51.1
KC40420-3	KBrB021L13	Ctg5	R01	51.9
KS40380	KBrB060L22	Ctg6	R01	53.0
KA40440-2	KBrB085D18	Ctg7	R01	58.3
KS40282	KBrH122A22	Ctg8	R01	61.6
KC40590-4	KBrB061L05	Ctg9	R01	65.8
KS50200	KBrB035A14	Singleton	R01	69.3
At4FCA	KBrH064P04	Ctg10	R01	72.7
BrFCA	KBrH051L05	Ctg10	R01	72.7
	KBrH064P04			
KS40660	KBrH009D02	Ctg11	R01	75.9
KC40560-1	KBrH001M22	Ctg12	R01	78.7
KS40551	KBrH003F06	Singleton	R01	79.1
KA40470-3	KBrB069A15	Singleton	R01	79.4
KR30650-1	KBrB048K11	Ctg13	R01	80.3
KS30540	KBrB042J11	Ctg14	R01	82.4
KR30480-1	KBrB082I19	Ctg15	R01	85.2
KR30462-1a	KBrH027N05	Ctg16	R01	86.2
KS30360	KBrB089M05	Ctg17	R01	93.3
KS30210	KBrH003N18	Ctg18	R01	119.1
KC30190	KBrB072P01	Singleton	R01	125.6
KS50320	KBrH015C19	Singleton	R02	14.4
KS50164_FLC2	KBrH004D11	Ctg19	R02	19.9
KS50070	KBrS003K07	Singleton	R02	30.4
KS50170	KBrB054O10	Ctg19	R02	38.9
KS50260	KBrB013N08	Ctg20	R02	49.2
KS50330	KBrB071M18	Singleton	R02	53.4
KS50410	KBrS004M05	Ctg21	R02	57.6
KS50920	KBrB049H14	Singleton	R02	67.2
KS50940	KBrB069F08	Ctg22	R02	67.7
KS50890	KBrB001N22	Ctg23	R02	71.2
KS50860	KBrB078H21	Ctg24	R02	73.8
KS50880	KBrB071K22	Ctg23	R02	75.6
KS11140	KBrB010O09	Ctg25	R02	78.9
KS11160b	KBrB005J17	Ctg26	R02	79.8
KR11300-1	KBrB084M08	Ctg27	R02	80.7
KR11290-1	KBrB034I14	Ctg28	R02	81.1
KS31020	KBrB084F02	Singleton	R02	82.6
KS20580d	KBrB052E10	Ctg29	R02	85.8
KS11120	KBrB070J11	Singleton	R02	86.9
KA40120-2	KBrB021M05	Singleton	R02	97.0

KR50776-1a	KBrH128M07	Ctg30	R02	104.1
KS10420	KBrB056F01	Ctg31	R02	112.4
KS51151_MAF1	KBrH080C09	Ctg33	R02	114.0
KS50460	KBrB086G22	Ctg32	R02	115.9
KS51090	KBrH014E13	Ctg36	R02	119.0
KS51140	KBrB007I19	Ctg33	R02	120.4
KS51180	KBrB021J09	Ctg34	R02	121.7
KA30820-2	KBrH004H18	Ctg35	R02	123.3
KS51115	KBrB066B03	Ctg36	R02	123.7
KS31050	KBrB073D09	Ctg37	R02	125.9
KS51100	KBrB036H12	Ctg36	R02	128.7
KR40830-1b	KBrB043B23	Ctg38	R03	0.0
KS20540b	KBrB074K06	Ctg65	R03	0.0
KS30660	KBrB084M06	Singleton	R03	46.2
KR40313	KBrH045E23	Ctg63	R03	48.2
KS40400	KBrB085J21	Ctg39	R03	48.9
KS30930	KBrB084F01	Singleton	R03	52.0
KS40270	KBrS003O10	Ctg63	R03	55.6
KS30920	KBrS012D09	Ctg51	R03	56.7
KR20080-1	KBrS008C11	Ctg48	R03	58.2
KS40250	KBrB089H07	Ctg47	R03	59.7
KS30380	KBrB052N08	Ctg49	R03	62.9
KS30370	KBrB010F13	Ctg49	R03	67.0
KR20070-1	KBrB058B22	Ctg48	R03	68.6
BAN276	KBrH097H02	Ctg52	R03	71.6
BAN293	KBrH024M10	Ctg53	R03	73.8
KS30463	KBrH054O10	Singleton	R03	76.2
KS30300	KBrB047D06	Ctg51	R03	76.8
KS30350	KBrB021F10	Singleton	R03	77.6
KS30500	KBrB044L16	Ctg55	R03	78.3
KS30310	KBrH001D20	Ctg51	R03	81.2
KS30251	KBrH122D17	Ctg56	R03	82.4
KC30030	KBrS007P17	Singleton	R03	85.2
KS30180	KBrB007J07	Ctg60	R03	89.4
KBrH065D07_BrCO4	KBrH065D07	Ctg61	R03	94.1
KS30040	KBrB001H24	Singleton	R03	96.3
BAN236	KBrH056G12	Ctg62	R03	100.5
KS20470	KBrB068E07	Singleton	R03	105.9
KS11150	KBrH011O17	Ctg1428	R03	108.9
BAN50	KBrH072P15	Ctg67	R03	119.0
KS10870	KBrB065E07	Ctg68	R03	119.6
KR20690b	KBrH007P05	Ctg69	R03	120.8
KR20690-2b	KBrH007P05	Ctg69	R03	120.8
KS20620	KBrS012M03	Ctg71	R03	123.5
KR20491_FLC5	KBrH038M21	Ctg72	R03	126.1
KS50980	KBrB071P24	Ctg74	R03	133.7
KS50870	KBrB054N05	Ctg76	R03	137.3
KS50360	KBrB004L02	Ctg1428	R03	139.1
KS50910	KBrH004B20	Ctg77	R03	139.6
KR50290-1	KBrB018H04	Ctg63	R03	147.7
KS50230	KBrB008I08	Singleton	R03	148.1
KS20520	KBrH005J02	Ctg81	R03	153.7
KS50270	KBrH003M07	Ctg82	R03	155.5

KS50300	KBrB016B21	Ctg63	R03	156.6
KR50161_FLC3b	KBrH052O08	Ctg87	R03	161.0
KR50163_FLC3a	KBrH117M18	Ctg86	R03	161.9
KS50140	KBrH006C14	Ctg87	R03	163.4
KS50090	KBrB055N13	Ctg88	R03	165.5
KR50010	KBrB045I17	Ctg90	R03	168.3
KS50050	KBrB092L14	Ctg91	R03	173.3
KS30982	KBrH006P22	Ctg95	R04	24.3
KS31030	KBrB056C05	Singleton	R04	25.1
KS20332	KBrH131C09	Ctg96	R04	36.7
KS20600	KBrB026E08	Ctg97	R04	57.6
KS20640	KBrB089M13	Ctg98	R04	66.5
KA20680-2	KBrS003G14	Singleton	R04	66.9
KS20730	KBrB089D07	Singleton	R04	70.9
KS20720	KBrB033O04	Ctg99	R04	71.8
KS20740	KBrH004D08	Ctg100	R04	73.7
KR20810-4	KBrH014A02	Ctg1428	R04	76.4
KS20840	KBrH009I04	Ctg1428	R04	78.6
KR20980-1	KBrS004I08	Singleton	R04	89.0
KS40410	KBrB067M19	Ctg101	R05	0.0
KR20951_BrPRR9	KBrB027C16	Ctg102	R05	5.4
KS20860	KBrB010H06	Ctg103	R05	19.4
KS20851	KBrH117N09	Ctg104	R05	22.2
KS20890	KBrH014M21	Ctg106	R05	36.1
BAN30	KBrH101P04	Ctg105	R05	39.7
KR20930-1	KBrB086L12	Ctg107	R05	46.3
KS20770	KBrB051I12	Ctg108	R05	48.5
KS20760	KBrB023F24	Singleton	R05	50.9
KS20710	KBrB082L07	Ctg106	R05	56.4
KS20700	KBrH015L23	Singleton	R05	64.2
KS20650	KBrB050K06	Ctg109	R05	66.7
KC40240-3	KBrB085G17	Ctg110	R05	78.8
KS11020	KBrH001D10	Ctg111	R05	86.3
KA30560-1	KBrB046C11	Singleton	R05	87.8
KS10850	KBrS012D15	Ctg112	R05	91.3
KS30340	KBrH001C24	Ctg113	R05	108.5
KS30270	KBrB039A19	Singleton	R05	120.5
KS30260	KBrB063G23	Ctg114	R05	125.1
KS30220	KBrB087P06	Ctg115	R05	128.7
KS30240	KBrH004P05	Ctg114	R05	130.1
KS30160	KBrB072L17	Singleton	R05	138.7
KA30150-2	KBrB042L19	Ctg116	R05	141.5
KS50180	KBrB020E06	Ctg117	R05	157.7
BrLEAFY-I	KBrH005P03	Ctg128	R06	0.0
KR10990-1	KBrS012H18	Ctg1428	R06	0.0
KS10980	KBrB022P06	Ctg119	R06	2.7
KS10970	KBrB018N18	Ctg120	R06	6.1
KS10950	KBrB065N20	Ctg121	R06	9.5
KS10190	KBrB086J10	Ctg122	R06	30.9
KS10280	KBrB086M23	Ctg123	R06	40.9
KC10310-2	KBrB034J13	Ctg124	R06	52.1
KS10321	KBrH134H15	Ctg125	R06	55.8
KR10390-1	KBrS007I12	Singleton	R06	68.2

KS10400	KBrB043O20	Singleton	R06	68.8
KS10410	KBrH006P24	Ctg126	R06	69.6
BAN295	KBrH030A15	Ctg129	R06	71.2
KS10450	KBrB018K09	Ctg129	R06	73.9
KS30830	KBrB044D19	Ctg127	R06	74.2
KS51082	KBrH005P03	Ctg128	R06	75.4
KS30840	KBrB062J16	Ctg130	R06	77.7
KS10460	KBrB011L09	Ctg129	R06	81.8
KS50480	KBrB023I24	Singleton	R06	85.0
KS50550	KBrS016J18	Ctg131	R06	95.4
KS30770	KBrH013N10	Ctg132	R06	102.9
KS30720	KBrB089H11	Singleton	R06	107.4
KS30740	KBrS004A14	Singleton	R06	111.5
KS20050	KBrH009H15	Singleton	R06	113.1
KR30750-2a	KBrH003P24	Ctg132	R06	116.5
KS11561	KBrH004M24	Ctg133	R06	120.0
KS50760	KBrB021P11	Singleton	R06	124.6
KS50740	KBrB034G01	Ctg134	R06	128.1
KS50720	KBrB007O13	Ctg134	R06	129.7
KS50700	KBrH003E13	Ctg134	R06	132.1
KR50690-2	KBrS016L08	Ctg134	R06	134.9
KS50670	KBrS008G07	Ctg135	R06	141.5
KS50630	KBrB061E18	Singleton	R06	143.3
KS20240	KBrH001J06	Ctg136	R07	10.0
KS10730	KBrS003D10	Singleton	R07	11.8
KS10570	KBrB080N15	Ctg137	R07	31.3
KR50440-1a	KBrB063C05	Ctg138	R07	32.4
KS31001	KBrH102A07	Ctg139	R07	57.2
KS31100	KBrB070L01	Ctg140	R07	66.1
KR31140-5	KBrB027O09	Ctg140	R07	68.8
KS11440	KBrB026G01	Ctg141	R07	79.7
KS11280	KBrB073K15	Singleton	R07	82.8
KS11130	KBrB083K19	Singleton	R07	85.6
KS11310	KBrB057E05	Ctg142	R07	85.8
KR11200-1	KBrB084K02	Ctg143	R07	88.5
KR11340-1a	KBrH011C10	Ctg144	R07	90.5
KS11170	KBrH010F15	Ctg145	R07	91.7
KS11250	KBrH012E04	Ctg146	R07	99.1
KS11351	KBrH031P22	Ctg147	R07	106.9
KS11390	KBrH011B08	Ctg148	R07	110.8
KS11360	KBrB086N06	Ctg147	R07	113.9
KR11490-1a	KBrB061K11	Ctg149	R07	115.6
KS11380	KBrB056L15	Singleton	R07	118.8
KR50040-2	KBrB036G18	Singleton	R07	120.3
KR10130-1	KBrB090M17	Singleton	R08	0.0
KR10140-2	KBrH001D23	Singleton	R08	17.1
KS10160	KBrB037O12	Ctg150	R08	20.2
KS10150	KBrH013B19	Ctg150	R08	24.5
KC10200-3	KBrH001D09	Singleton	R08	25.8
KS10240	KBrB006B05	Ctg151	R08	27.7
KS10220	KBrB080I02	Ctg152	R08	28.0
KS10250	KBrB017B11	Singleton	R08	34.5
KS10320	KBrB077F22	Singleton	R08	36.7

KS10440	KBrB042N05	Ctg154	R08	39.3
KR10610-1a	KBrB052E19	Ctg153	R08	41.2
KS10550	KBrB006C05	Ctg155	R08	46.9
KR10430-1	KBrB058M10	Ctg154	R08	51.7
KR10380-2	KBrB044C04	Ctg156	R08	53.1
KC10680-1	KBrB016E20	Ctg157	R08	63.9
KS10710	KBrB027K16	Ctg157	R08	66.5
KR40760-3	KBrB048L11	Ctg158	R08	67.9
KS40981	KBrH004J18	Ctg159	R08	72.4
KS40930	KBrH005C21	Ctg160	R08	74.9
KR40700-1	KBrB006O19	Ctg161	R08	80.0
KS40480	KBrB075L03	Ctg162	R08	83.2
KS40490	KBrB006A15	Ctg162	R08	84.4
KS40300	KBrB056G23	Singleton	R08	88.4
KS40314	KBrH138O03	Ctg163	R08	89.4
KS40340	KBrS008P19	Ctg164	R08	93.7
KS11010	KBrH013K13	Ctg165	R08	98.6
KC40580-4	KBrB059G16	Ctg166	R08	99.4
KS10750	KBrB012O24	Singleton	R08	102.0
KS40210	KBrB019A15	Singleton	R08	102.6
KS10110	KBrB012F17	Ctg168	R09	0.0
KS10050	KBrB043F18	Ctg167	R09	21.6
KC10122-1	KBrH010M08	Ctg169	R09	29.3
KS10020	KBrB092L06	Singleton	R09	30.7
KS10040	KBrB089B13	Ctg167	R09	33.5
KS10121	KBrH138P04	Ctg168	R09	39.8
BAN66	KBrH138P04	Ctg168	R09	44.1
KR10230-3	KBrB044J13	Ctg171	R09	46.7
KC10350-3	KBrB023K01	Ctg172	R09	65.6
KC20280-2	KBrB082F21	Ctg174	R09	68.5
BAN286	KBrH032H04	Ctg175	R09	69.8
KS20291	KBrH081N08	Singleton	R09	74.7
KS20300	KBrB019I24	Ctg176	R09	77.7
KS20310	KBrB025M01	Ctg176	R09	82.3
KS20390	KBrB063K02	Ctg179	R09	84.6
KS31203	KBrH001H24	Ctg180	R09	86.0
BAN2	KBrH049F16	Singleton	R09	88.8
BAN235	KBrH071P14	Ctg180	R09	89.2
KS31191	KBrH076J01	Ctg180	R09	90.1
BIF30	KBrH042F19	Singleton	R09	90.5
KS31180	KBrB044E18	Ctg181	R09	91.8
KS31150	KBrB036H01	Singleton	R09	92.1
KS31201	KBrH042F19	Singleton	R09	93.7
KS31002	KBrH117O12	Ctg1428	R09	98.3
BIF77b	KBrH117O12	Ctg1428	R09	99.5
KS10551	KBrH015M19	Ctg190	R09	104.8
KS30880	KBrB049N17	Ctg191	R09	106.0
BIF80a	KBrH107E12	Ctg193	R09	108.5
KR10540	KBrB048C04	Ctg194	R09	110.3
KS30900	KBrB088I08	Singleton	R09	111.3
KS10621	KBrH143H14	Ctg198	R09	114.5
KS10630	KBrS010I09	Singleton	R09	114.7
KS40620	KBrB028P01	Ctg197	R09	115.6

KBrH013B21	KBrH013B21	Ctg200	R09	116.7
BIF5	KBrH143H14	Ctg198	R09	117.1
KC10590-1	KBrB066M16	Ctg201	R09	117.5
KS10600	KBrB059J21	Singleton	R09	118.7
KS10840	KBrB063M04	Singleton	R09	119.7
KS50730	KBrB034P04	Ctg203	R09	124.6
KS20030	KBrB072E02	Ctg204	R09	124.8
BAN40b	KBrH085A21	Ctg205	R09	125.7
KS50774	KBrH077A05	Singleton	R09	126.8
KS11050	KBrB037F09	Ctg207	R09	127.5
KS11040	KBrB086C10	Ctg207	R09	128.7
KS50621	KBrH099I08	Ctg208	R09	129.5
BIF81a	KBrH099I08	Ctg208	R09	130.4
KS50660	KBrB053J19	Ctg209	R09	133.9
KS11100	KBrB025K04	Ctg211	R09	135.2
KS10760	KBrB016K20	Ctg212	R09	137.7
BIF129a	KBrH106L05	Singleton	R09	138.8
KS11090	KBrB069A23	Singleton	R09	142.6
KS20260	KBrH014M07	Ctg215	R09	144.9
KBrH115B11	KBrH115B11	Ctg218	R09	152.8
KS51170	KBrB011P07	Ctg216	R09	159.7
KS51160	KBrB022L12	Ctg217	R09	167.4
KS51050	KBrB051M06	Ctg219	R09	175.4
KS50470	KBrB068N22	Ctg218	R09	175.6
KS50490	KBrB005E24	Ctg218	R09	176.6
KS50522	KBrH098A19	Singleton	R09	178.7
KS50524	KBrH125N23	Ctg224	R09	179.4
KS50521	KBrH133J19	Ctg224	R09	180.1
KS50775	KBrH067N03	Ctg221	R09	181.1
KS51130	KBrB031O20	Ctg222	R09	182.6
KS30763	KBrH097M21	Ctg223	R09	183.9
BAN99	KBrH085N21	Ctg223	R09	192.6
BAN56	KBrH097M21	Ctg223	R09	193.9
KR40090-2	KBrB037E04	Ctg1428	R09	198.0
BIF94b	KBrH123K18	Ctg227	R09	202.0
BAN219	KBrH121P05	Ctg228	R09	203.7
BAN259b	KBrH125N23	Ctg224	R09	213.2
KS50166_FLC1	KBrH080A08	Ctg233	R10	11.8
KS50150	KBrB034N10	Ctg233	R10	12.9
KS50190	KBrB080E24	Ctg234	R10	29.5
KS50240	KBrH009B23	Ctg235	R10	32.0
KS50310	KBrB087B10	Ctg236	R10	36.3
KS50340	KBrB015N02	Ctg237	R10	47.7
KS11510	KBrB006F18	Ctg238	R10	49.9
KS50390	KBrB067F22	Ctg239	R10	52.4
KS50420	KBrH012D09	Singleton	R10	53.2
KS50990	KBrB043M07	Ctg240	R10	54.6
KS50450	KBrB036L21	Singleton	R10	56.9
KS51021	KBrB010B23	Ctg241	R10	59.2
KS10091	KBrH053G06	Singleton	R10	61.6
KS50640	KBrH013D23	Ctg242	R10	72.3
KS10051	KBrH003L04	Singleton	R10	73.9
KR10010-2	KBrB046G18	Singleton	R10	87.8
