# Supplementary material for: The first generation of a BAC-based physical map of Brassica rapa
Source: BMC Genomics. 2008 Jun 12;9:280. doi: 10.1186/1471-2164-9-280 (PMC2432078; doi:10.1186/1471-2164-9-280)
Supplement: Additional file 2 — Figure S1. The clone order fingerprints of 19 of 22 BAC clones for the BAN2 marker. This figure shows fingerprinted band image of 19 positive BAC clones of the BAN2 marker. [file 1471-2164-9-280-S2.ppt]

## Slide 1
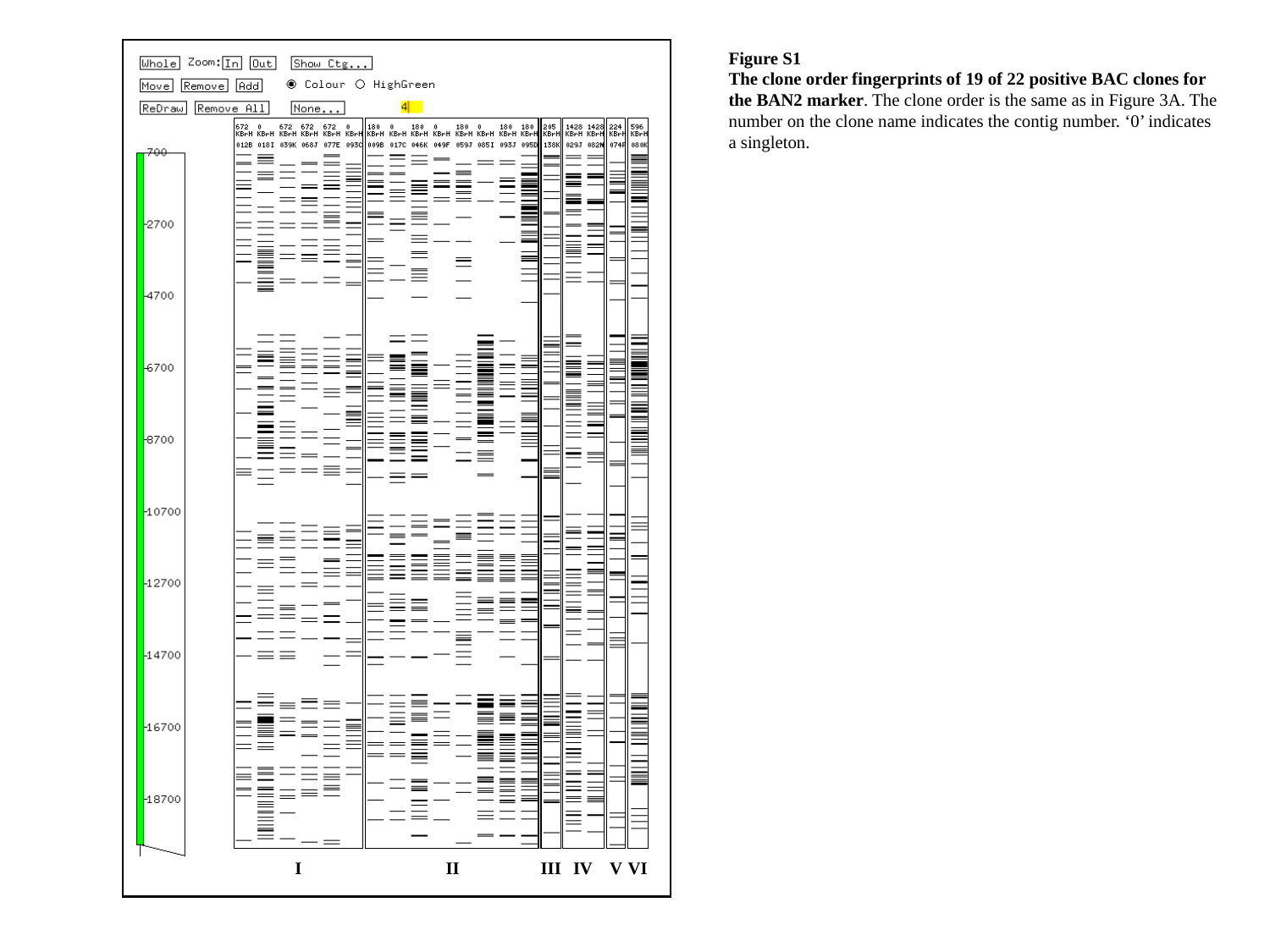

Figure S1
The clone order fingerprints of 19 of 22 positive BAC clones for the BAN2 marker. The clone order is the same as in Figure 3A. The number on the clone name indicates the contig number. ‘0’ indicates a singleton.
I
II
III
IV
V
VI
